# Assessing Pedophilic Sexual Interest: The Utility of Objective Correlates in Diagnostic Decisions

**DOI:** 10.5964/sotrap.14917

**Published:** 2025-07-15

**Authors:** Raquel Yeomans, Drew A. Kingston

**Affiliations:** 1 HOPE Program, San Diego, CA, USA; 2 Department of Psychiatry, McMaster University, Hamilton, ON, Canada; Saint Mary's University, Halifax, NS, Canada

**Keywords:** pedophilic sexual interest, paraphilias, sexual offending, assessment, behavioral correlates

## Abstract

Pedophilic sexual interest is a risk factor for sexual recidivism and has particular relevance for a significant proportion of individuals who have sexually offended against a child. In this article, we review the predominant ways in which pedophilia is assessed, including both direct and indirect approaches. The limitations of these methods are highlighted and the utility of incorporating more objective indicators into a comprehensive evaluation is examined. Particular emphasis is placed on the inclusion of behavioral correlates into a comprehensive and multimodal assessment approach. Implications for the assessment of individuals convicted of sexual offending and who present with pedophilic sexual interest are discussed.

## Highlights

- Summarizes the importance of pedophilic sexual interest in the understanding of sexual offending.
- Reviews the predominant approaches towards assessment of pedophilic sexual interest along with some notable limitations.
- Reviews some preliminary evidence for the value of incorporating behavioral correlates in making diagnostic decisions related to pedophilic sexual interest.

Pedophilia is defined as a sexual interest, or preference^1^1Although interest and preference are sometimes used interchangeably, the former underscores an absolute sexual response to children whereas the latter denotes whether that response is stronger than the response to adults (Schippers et al., 2023; Schmidt et al., 2017). We utilize the term interest throughout this paper., for prepubescent children, which can be manifested by recurrent sexually arousing fantasies, sexual urges, or behaviors involving sexual activity with children (American Psychiatric Association [APA], 2013). In the DSM-5, pedophilic interest can be diagnosed as Pedophilic Disorder when the sexual interest results in marked distress, interpersonal difficulty, or when the individual has acted on this interest with a child (APA, 2013). Although 13 years of age and younger is the generally accepted age cut-off for prepubescence, this is meant as a proxy for the various stages of pubertal development (see Sexual Maturation Scale; Marshall & Tanner, 1969). Of note, increased attention has been directed toward hebephilia, which describes an erotic age interest in pubescent children or those in the early stages of secondary sexual development (Blanchard, 2010; Stephens et al., 2017). Previous studies have suggested that an interest in pubescent children is relatively distinct from an interest in prepubescent children (Blanchard et al., 2009; Stephens & Seto, 2016); however, more recent studies have underscored some overlap between these two groups (Stephens et al., 2019). Pedohebephilia was proposed for inclusion in the DSM-5, but was rejected for inclusion with only pedophilia, or Pedophilic Disorder, being formally recognized.

Although an individual may meet the criteria for Pedophilic Disorder, or express pedophilic sexual interest, this does not necessarily mean that they will engage in sexual activity with a child (Cantor & McPhail, 2016); alternatively, some individuals who have engaged in sexual acts with children do not have sexual interest in children. Pedophilia is, however, one of the most commonly reported paraphilias among individuals in correctional or forensic settings and, in fact, is the most cited paraphilia diagnosis in civil commitment hearings (Lu et al., 2015; McLawsen et al., 2012). Unfortunately, several concerns have been raised regarding the assessment of pedophilia and the utility of this diagnosis in high-stakes proceedings.

In this paper, we review the predominant methods in which pedophilia is assessed and note some important limitations. We discuss the value and utility of incorporating objective indicators, particularly behavioral correlates, in a comprehensive multimodal assessment of this construct. This may have promising implications for both the reliability and validity of psycho-legal evaluations for those who have committed sexual offenses against children and in which potential sexual interest in children is a concern.

## Pedophilia and Sexual Offending

The prevalence of pedophilia has, in general, been based on selected clinical samples; that is, those who have acted on their interests and have been charged or convicted of a sexual offense. Some research, however, has included non-clinical population-based samples. Prevalence rates for pedophilia in the general population have been estimated between 1% and 5% (Bártová et al., 2021; Dombert et al., 2016; Joyal et al., 2015; Santtila et al., 2015; Winters et al., 2023). For individuals who have offended against a child, these rates have been observed between 27% and 70% (Schmidt et al., 2013; Seto & Lalumière, 2001; Wilson et al., 2011). Such variability is likely due to differences across studies, such as sample composition, assessment, and diagnostic methodology. Despite the variable prevalence rates, pedophilic interest is clearly over-represented among those who offend against children compared to other samples. Pedophilic sexual interest is also a salient factor with relevance for both the onset and persistence of sexual offending (Seto, 2008).

A sexual interest in children has been included in various theories proposed to explain the occurrence of sexually aggressive behavior. For example, Lalumière and Quinsey (1994) presented their Sexual Preference Hypothesis that underscored the notion that men engage in sexually aggressive acts because they prefer this behavior over consensual sexual activity. While intuitively appealing and applicable to a certain proportion of men who engage in sexual offending, the theory does not account for all instances of sexual offending.

Ward and Beech (2006) provided an expansive explanation of sexual offending that included paraphilias as one component of the model. The Integrated Theory of Sexual Offending (ITSO) included neurological, biological, and ecological factors to understand sexual offending against both adult and child victims. The authors suggested that difficulties with one’s motivation/emotional system, as well as perception and memory system, may manifest as dynamic risk factors for sexual offending. These may include deviant sexual interests, including those involving children.

In another comprehensive account of sexual offending, Seto (2008, 2019) proposed the Motivation Facilitation Model (MFM) of sexual offending. The MFM underscores the interaction between motivation factors (e.g., paraphilic sexual interest) and facilitation factors, including trait factors (e.g., self-regulation problems, antisocial personality), state factors (e.g., substance use, negative affect), and situational factors (e.g., access to victims, presence of a guardian, location, time), to explain the antecedents to sexual offending. In support of the MFM, Klein and colleagues (2015) discovered that high sex drive (i.e., motivation factor), sexual fantasies involving children (i.e., motivation factor), and antisocial personality (i.e., trait factor) were associated with consumption of child sexual exploitation material (CSEM). Additionally, a study conducted in Hong Kong found that interest in zoophilia and exhibitionism increased the tendency to engage in nonpenetrative and penetrative sexual assault, but decreased nonpenetrative-only sexual assault for those interested in zoophilia and voyeurism (Chan & Myers, 2023).

Pedophilic sexual interest has also been identified as an important factor in the persistence of sexual offending. Indeed, pedophilic sexual interest has shown to be strongly associated with sexual recidivism (Hanson & Morton-Bourgon, 2005), and has been identified as a psychologically meaningful risk factor (Mann et al., 2010). A meta-analysis by Schippers and colleagues (2023) reported that individuals with pedophilia who sexually offended against children had higher absolute sexual interest in children and lower sexual interest in adults compared to individuals who had never offended against a child. Given the importance of pedophilia as a predictor of sexual offending for some individuals, valid assessment methods are vital to ensure that risk level can be ascertained, and a treatment plan can be developed.

## Assessment of Pedophilia and Pedophilic Disorder

There are several ways in which to assess the sexual interests of individuals suspected of having pedophilic sexual interest (see Kalmus & Beech, 2005; Seto, 2018 for a review). Evaluators typically employ either direct measures of sexual interest (e.g., interview/questionnaires) that predominantly rely on self-report, or indirect measures that are either physiologically based (e.g., plethysmograph) or based on attentional or other proposed mechanisms (e.g., viewing time, information processing measures).

### Direct Measures of Sexual Interest

Sexual interest can be inferred from a comprehensive clinical interview, whereby respondents are asked questions pertaining to their sexual thoughts, interests, and behaviors. Interviews can be informative, particularly when a respondent admits to sexually inappropriate thoughts and behaviors. Despite this, interviews are subject to socially desirable responding and many individuals convicted of sexual offending deny or minimize certain aspects of their sexual histories (Dietz, 2020).

Despite the potential for socially desirable responding, directly asking an individual about their sexual interests is the most straight-forward way to gather information. Details about past offenses and sexual behavior (e.g., pornography use) are often asked, and questions typically correspond with established DSM-5 or ICD-11 criteria. As noted earlier, diagnostic criteria are the guiding standard with which Pedophilic Disorder is determined, and it is widely used in psychosexual evaluations, particularly in high-stakes settings such as civil commitment. Of course, information obtained from a clients’ self-report is largely influenced by the context of the assessment; that is, individuals may be inclined to respond more accurately if they self-refer for concerns about a sexual interest in children compared to those already in the criminal justice system and who are mandated to attend treatment. The limitations of self-report, and associated clinical judgment, have potential ramifications for the utility of nosological diagnosis (Hanson & Morton-Bourgon, 2009; Harris et al., 2015).

Several studies have underscored potential problems and an overall lack of utility of diagnostic formulation, particularly among individuals who have sexually offended (Marshall, 2006; Marshall & Kingston, 2018; Moser & Kleinplatz, 2020). Marshall and Kingston (2018) summarized several problems with the paraphilic disorders as a proper nosological system, as well as issues with the defining features of the diagnoses and lack of precision of the criteria.

A central criticism of the diagnosis of pedophilia, among other paraphilic diagnoses (e.g., sexual sadism; see Kingston & Yates, 2008; Marshall & Kennedy, 2003; Nitschke et al., 2013), is that the criteria are vague and subjective. Indeed, the terms “recurrent” and “intense” necessitate a considerable degree of clinical judgment that is based on self-reported information, which, in many cases, is questionable. The limitations and fallibility of clinical judgment are well-documented (Hanson & Bussière, 1998; Meehl, 1954; Quinsey et al., 2006). Moreover, the requirement for the interest to cause “distress” or “impairment” raises questions regarding its applicability to all individuals that experience pedophilic sexual interest. For example, some individuals may experience negative emotions and be impaired by their interests, but others may not, such as when distress results from being arrested, or among those whose behavior is ego-syntonic (e.g., members of pedophilic organizations). These concerns are likely significant causes of the problems with both the reliability and validity of the diagnoses.

Packard and Levenson (2006) found that diagnoses that are less common (e.g., less familiar to the raters) and with relatively vague diagnostic criteria had poorer interrater agreement. Other studies have also demonstrated that these levels fall below that which can support a useful guide for diagnostic purposes (Levenson, 2004; Marshall & Kingston, 2018). In fact, Mokros and colleagues (2018) found that 1 in 3 pedophilia diagnoses may be inaccurate after examining interrater agreement.

Much of the research addressing risk predictors and recidivism rates among individuals deemed to be pedophilic has been extrapolated from studies with participants who have offended against children and who may or may not have met the diagnostic criteria for pedophilia. Among those diagnosed with pedophilia, studies have generally revealed poor predictive accuracy for psychiatric diagnosis, a finding that has been replicated with other paraphilias, such as sexual sadism (see Kingston et al., 2010; cf. Eher et al., 2015). Kingston and colleagues (2007) evaluated the utility of the diagnosis of pedophilia in a sample of 206 individuals convicted of sexual offenses against children. Pedophilia was determined based on psychiatric diagnosis, phallometrically assessed sexual arousal to children, various behavioral indicators (Seto & Lalumière, 2001), and a combination of the above correlates. Results revealed few statistically significant differences between those deemed to be pedophilic versus non-pedophilic, regardless of the classification system used, and was particularly poor when using the DSM. Importantly, no differences were evident on features considered important in assessing risk of recidivism, including criminal history, victim characteristics, and marital status. Finally, it was concerning that DSM-based diagnoses were not associated with other classification methods.

In a follow-up study using the same methodological approach toward classification, Moulden and colleagues (2009) directly examined the predictive utility of the diagnosis of pedophilia on recidivism assessed up to 20-years post-release. Results were consistent with earlier studies demonstrating concerns with the validity and utility of the diagnosis of pedophilia. A DSM diagnosis of pedophilia revealed particularly poor predictive accuracy and discriminant validity among recidivists and non-recidivists, especially when compared to more objective correlates (i.e., phallometrically assessed sexual arousal to children). In another example, Wilson and colleagues (2011) examined a sample of individuals convicted of sexual offending who were assessed using phallometric testing, DSM-IV diagnoses, and the Rapid Risk Assessment of Sex Offender Recidivism (RRASOR; Hanson, 1997). Results indicated inconsistencies in the diagnosis of pedophilia, and that only the RRASOR was predictive of sexual recidivism.

Given the complications with nosological diagnoses, and the concerns regarding self-report more generally, increased attention has been made on incorporating measures that are less susceptible to impression management and with less reliance on subjective interpretation of somewhat vague criteria. It is conceivable that utilizing methods that are less susceptible to impression management and clinician judgment would produce findings that may be more beneficial for risk management and treatment planning.

### Indirect Measures of Sexual Interest

#### Physiological Assessment

One of the more common methods of assessing sexual interest of people convicted of sexual offenses against children and adults is penile plethysmography, also referred to as phallometry or PPG (Barker & Howell, 1992). PPG involves the objective measurement of erectile changes in response to stimuli that varies on the dimension of interest, such as the age and sex of the individual depicted, or the level of coercion/violence during the sexual act.

PPG has been shown to have accurate sensitivity (Blanchard et al., 2001; Freund & Watson, 1991), discriminant validity (Harris et al., 1992), predictive validity (Hanson & Bussière, 1998; Hanson & Morton-Bourgon, 2005), and convergent validity with other approaches to assessing pedophilic sexual interest (Schmidt et al., 2017). Moreover, a recent meta-analysis showed that tests using slide and audio, but not video, stimuli predicted sexual recidivism (McPhail et al., 2019). Nevertheless, there is evidence that PPG can be influenced by low responding (O’Donohue & Letourneau, 1992), with some concerns pertaining to an overall lack of reliability (Barbaree & Marshall, 1989) and validity (Hall et al., 1988). Moreover, it has been reported that some individuals are able to inhibit their reported arousal by suppressing penile responses (Howes, 1998; Kalmus & Beech, 2005; Marshall & Fernandez, 2000); although, in one study this was not more than what would be expected by measurement error (Babchishin et al., 2017). In addition, not all individuals will be aroused by the stimuli presented, but may be aroused by stimuli that is not depicted (Bahroo, 2003). Finally, a major problem with PPG is the lack of standardization in testing procedures and protocol across labs and countries (see Murphy et al., 2015).

#### Latency-Based Measures

There are a few other measures that are based on more implicit mechanisms that were intended to minimize the limitations noted earlier (Banse et al., 2010; Kalmus & Beech, 2005; Seto, 2008). Such modalities have been based on viewing time or other cognitive/informational processing mechanisms.

Viewing time measures (VT) were developed from several proposed underlying mechanisms that ultimately impact latency when viewing potentially sexually arousing stimuli (see Wilson & Miner, 2016 for a review). First, viewing time was based on the simple notion that individuals will inherently view attractive images longer than images they find to be unattractive or neutral (Rosenzweig, 1942). Second, sexual content-induced delay (SCID; Imhoff et al., 2010) was proposed, whereby the latency is impacted when sexual content is introduced into a specific cognitive task. In other words, the presence of sexually arousing stimuli causes hesitancy in decision making (see Geer & Bellard, 1996). Finally, longer latencies could also be due to the task demands; that is, the ease in which attractive versus unattractive stimuli are affirmed or rejected (Imhoff et al., 2012).

In an examination of these mechanisms, Imhoff and colleagues (2012) found a task-driven effect as well as a stimulus-based effect while using VT measures, providing further evidence that VT may not rely solely on viewing time to assess one’s sexual attraction to stimuli. Fromberger and colleagues (2013) measured entry time and relative fixation time for individuals with pedophilic sexual interest and those without. The pedophilic participants showed shorter entry time, suggesting that individuals with pedophilic interest viewed children because they found them to be the most sexually relevant stimuli. Schmidt and colleagues (2021) examined stimulus-based processes versus cognitive tasks-based processes to predict VT effects and found evidence for prolonged response latencies, which was likely due to the tasks involved in determining sexual attractiveness.

VT measures have provided some useful information in risk and treatment planning and have shown generally good psychometric properties. Affinity, a VT assessment of sexual interest, has demonstrated internal consistency and reliability within the range sufficient for research purposes (Mokros et al., 2013; Worling, 2006). VT has also demonstrated appropriate levels of convergent validity via the relationship between self-report, PPG, and the Screening Scale for Pedophilic Interests (SSPI; Schmidt et al., 2017). With regard to group discrimination, several earlier studies noted that VT adequately differentiates between those with child victims and controls (Babchishin et al., 2014; Gress et al., 2013; Harris et al., 1996); however, in a more recent study using an online sample of men, VT and self-report ratings of images were not able to discriminate between individuals with and without a history of sexual offending (Pezzoli et al., 2022), although a history of sexual offending against children does not necessarily indicate a preference or interest in children. With regard to predictive validity, Gray and colleagues (2015) utilized Visual Reaction Time™ (VRT) to examine the reactions of men with varying sexual deviations, including those who had previously offended against a child. VRT showed that those with higher levels of sexual interest in children were more likely to sexually offend in the future.

Despite the potential benefits of VT, concerns related to individuals falsifying sexual interest remain. Indeed, once the underlying mechanism of latency is known, it is not difficult to create a non-valid response (Veas, 2015). In fact, some websites have dedicated pages to help individuals understand how VT assessments work, which subsequently alters the results of these assessments (see “Explaining the Abel Assessment,” 2024).

The Implicit Association Test (IAT), another latency-based measure originally developed by Greenwald and colleagues (1998), was designed to measure ones underlying automatic associations by having participants associate different categories together (e.g., child and adult, sexy and not sexy). The IAT was later altered to measure the child-sex association (CSA-IAT) of individuals who had offended against a child and those who had offended against an adult or had committed a violent offense (Gray et al., 2005). Gray and colleagues (2005) found that offenders with child victims associated children and sex in the IAT. Additionally, the CSA-IAT showed moderate sensitivity. IAT measures of sexual interest in children have shown group discrimination (Babchishin et al., 2013), convergent validity (Babchishin, 2009; Babchishin et al., 2013), and moderate sensitivity (Gray et al., 2005).

Other latency-based measures involve stimuli triggering attentional processing. For example, the emotional Stroop task (Williams et al., 1996) requires showing words and colors to participants who are then asked to name the color of the word. The latency of response should match the processing of the meaning of the word shown. To examine deviant sexual interest such as pedophilic interest with the emotional Stroop task, delayed response is examined in individuals using sexually themed words. The emotional Stroop task has shown group discrimination (Price et al., 2013; Smith & Waterman, 2004) and convergent validity, but only for male participants (Spada & Jeglic, 2015).

Overall, the approaches toward assessment noted earlier provide some potentially useful information. Unfortunately, some limitations remain, such as the ease in which VT results are manipulated once the underlying mechanism is known. As such, researchers have pointed to the potential value of looking at certain behavioral characteristics that could reliably indicate the presence of pedophilic sexual interest, regardless of the motivation of the respondent or potential bias from the evaluator.

### Behavioral Correlates

Information obtained from an individual’s past behavior, including sexual offending, can be useful in an assessment as it doesn’t rely on self-report or criteria that necessitates subjective clinical judgment. Past behavior is also less associated with problems related to implicit measures of pedophilic interest. The primary limitation when using behavioral correlates is the quality of the information from which the correlates are drawn from and the fact that some behaviors that have occurred may not have been detected.

Several relevant victim characteristics associated with pedophilic interest have been observed among individuals with child victims; these include, having multiple victims (Blanchard et al., 2001; Hall & Hall, 2007; Hanson & Bussière, 1998), younger victims (Herman-Giddens et al., 1997, 2012), male victims (Hall & Hall, 2007; Hanson & Bussière, 1998), and extrafamilial victims (Hanson & Bussière, 1998; Seto et al., 1999). These variables have since been subsumed with other relevant indicators within more structured assessments that can be scored to inform diagnostic decisions. These measures are described below and the shared criteria across measures are shown in Table 1.

**Table 1:** Behavioral Correlates and Offense/Offender Characteristics Across Measures

Characteristics	SSPI	SSPI-2	CASIC	SSPC
Male Victim(s)	*✓*	*✓*		
Number of Victims	*✓*	*✓*		*✓*
Younger Victim(s)	*✓*	*✓*		*✓*
Extrafamilial Victim(s)	*✓*	*✓*		
CSEM Possession/Conviction		*✓*	*✓*	*✓*
Confidence Approach				*✓*
Anal Penetration				*✓*
Direct Threats				*✓*
Marital History			*✓*	
Volunteer History			*✓*	
Online Sexual Communications			*✓*	
Number of Sexual Offenses				*✓*

#### The Screening Scale for Pedophilic Interests

The Screening Scale for Pedophilic Interests (SSPI; Seto & Lalumière, 2001), and the revised version of the scale (SSPI-2; Seto et al., 2017), are considered brief actuarial screening measures of pedophilic sexual interest. The SSPI included four items: (1) a male victim under the age of 15, (2) two or more victims under the age of 15, (3) at least one victim under the age of 12, and (4) at least one extrafamilial victim, with the additional item, (5) possession of CSEM, being incorporated into the revised version of the scale. Seto and Lalumière (2001) noted that individuals could score from 0 to 5 with a median score of 3 in the development sample. Individuals with a score of 5 were four times more likely to show greater penile response to children than to adults when compared to participants with a SSPI score of 0. Helmus and colleagues (2015) examined 410 adult men who were on community supervision and found a significant relationship between SSPI scores, the deviant sexual preference item of the Stable-2007 (Hanson et al., 2007), and sexual recidivism. They further noted that the SSPI was a viable proxy for the deviant sexual preference item on the Stable-2007, which measures an individual’s sexual interest in children. Additionally, Seto and colleagues (2017) examined convergent validity of the SSPI-2 among 1,900 participants assessed at the Centre for Addiction and Mental Health in Toronto, Canada. Participants were measured using PPG and were interviewed via semi-structured interviews. Results indicated that the SSPI-2 items were positively and significantly correlated with the phallometric index of sexual arousal to children, thus indicating that the SSPI-2 could be a proxy for PPG testing, and ultimately for assessing an underlying sexual interest in children.

#### The Correlates of Admitted Sexual Interest in Children

The Correlates of Admitted Sexual Interest in Children (CASIC; Seto & Eke, 2017) was developed as a substitute for item 5 on the Child Pornography Offender Risk Tool (CPORT; Seto & Eke, 2015). The items included are: (1) the offender never having been married, (2) possession of CSEM videos, (3) possession of CSEM stories, (4) evidence of CSEM interest for two or more years, (5) a history of volunteering in a role with access to children, and (6) communicating with a minor, or an officer posing as a minor, online. Seto and Eke (2017) included 286 participants with a CSEM conviction in Ontario, Canada and found that CASIC scores were significantly associated with admission of sexual interest in children. Additionally, Eke and colleagues (2019) found that substituting the CASIC for item 5 on the CPORT did not decrease predictive performance. Among a sample of individuals with a CSEM conviction, Von Franqué and colleagues (2023) found that the CPORT with a CASIC rating were able to predict recidivism while a clinical diagnosis was unable to do so.

#### The Screening Scale of Pedophilic Crime Scene Behavior

The Screening Scale of Pedophilic Crime Scene Behavior (SSPC; Dahle et al., 2014) was created to inform the assessment of pedophilic sexual interest. Dahle and colleagues (2014) included 113 opinions from expert witnesses to determine the items on the SSPC. The scale includes the following items: (1) more than two victims under the age of 14, (2) 11 or more separate offenses against a victim or victims under the age of 14, (3) a victim who was 9-years-old or younger, (4) possession of CSEM, (5) evidence of trust building (e.g., grooming), (6) anal penetration, and (7) the use of direct threats with the victim or victims (reverse-scored item). Total scores of 5 and above are considered pedophilic. Lehmann and colleagues (2022) examined scores on the SSPC, PPG results, and clinical assessments from 316 individuals with child victims who were referred to the Massachusetts Treatment Center for evaluations. Results showed that the SSPC demonstrated significant diagnostic accuracy, incremental validity, and convergent validity.

## Conclusions and Future Implications

Pedophilic sexual interest is an important factor to consider when assessing an individual in correctional or forensic mental health settings. The estimated prevalence of pedophilia in the general population is relatively low (Joyal et al., 2015; Santtila et al., 2015; Winters et al., 2023), but is markedly over-represented among those who have committed a sexual offense, particularly against a child (Schmidt et al., 2013; Seto & Lalumière, 2001; Wilson et al., 2011). The reliable and valid assessment of this construct is particularly important due to the fact that a sexual interest in children is influential, both for the etiology of sexual offending and for its potential recurrence (Hanson & Morton-Bourgon, 2005; Mann et al., 2010; Seto, 2008).

Predominant assessment options discussed earlier include both direct and indirect approaches. Despite their wide-spread use, notable concerns remain for DSM-based diagnostic decisions (Kingston et al., 2007; Marshall & Kingston, 2018; Moulden et al., 2009) and, to a somewhat lesser extent, PPG (Bahroo, 2003; Howes, 1998; Kalmus & Beech, 2005; Marshall & Fernandez, 2000) and VT (Veas, 2015).

Behavioral correlates have shown considerable promise in the assessment of pedophilic sexual interest, and several measures are now available which combine these variables in a structured assessment. The advantage of these tools are that they are less susceptible to deliberate manipulation by the participant or any inherent clinician bias. Of course, the utility of these correlates are based on the availability of this information in the file and one cannot account for behaviors that are not included in collateral documentation, either due to these behaviors not occurring or going undetected.

Overall, incorporating behavioral correlates into a broad and multimodal assessment battery will increase the likelihood of meaningful diagnostic decisions essential for risk management and treatment planning. Others have emphasized the utility of a multimethod approach to assessing sexual interest, and more specifically, the value of including behavioral correlates (see Kalmus & Beech, 2005). Banse and colleagues (2010) explored the development and utility of the Explicit and Implicit Sexual Interest Profile, a multimethod approach to examining sexual interest in children. This tool included self-report as well as different iterations of IAT and VT methodologies. Results indicated that the tool was both reliable and valid. Although specific approaches to the assessment were better in isolation, the integration of measures resulted in improved predictive validity.

Sexual interest and its relationship to behavior is a complex phenomenon with potentially serious implications for the client. Any approach aimed at identifying this profile should be comprehensive and multimodal. Integrating measures that are conceptually coherent but, importantly, provide incremental validity should provide meaningful data with which an evaluator can make an informed decision regarding diagnoses and treatment planning. Measures that result in a coherent picture are informative but, so too, are data points that underscore important differences. Again, the evaluation would be more fruitful when based on varying sources of information while acknowledging the strengths and limitations of each approach.

## Social Media Accounts

*Drew A. Kingston:* X / Twitter
